# ΔFosB is part of a homeostatic mechanism that protects the epileptic brain from further deterioration

**DOI:** 10.3389/fnmol.2023.1324922

**Published:** 2024-01-12

**Authors:** Jerome Clasadonte, Tania Deprez, Gabriel S. Stephens, Georges Mairet-Coello, Pierre-Yves Cortin, Maxime Boutier, Aurore Frey, Jeannie Chin, Marek Rajman

**Affiliations:** ^1^Epilepsy Discovery Research, UCB Biopharma SRL, Braine-l’Alleud, Belgium; ^2^Baylor College of Medicine, Houston, TX, United States

**Keywords:** ΔFosB, granule cell dispersion, neuroinflammation, AAV-shRNA-ΔFosB, mossy fibers, homeostatic mechanism, mouse pilocarpine model of epilepsy

## Abstract

Activity induced transcription factor ΔFosB plays a key role in different CNS disorders including epilepsy, Alzheimer’s disease, and addiction. Recent findings suggest that ΔFosB drives cognitive deficits in epilepsy and together with the emergence of small molecule inhibitors of ΔFosB activity makes it an interesting therapeutic target. However, whether ΔFosB contributes to pathophysiology or provides protection in drug-resistant epilepsy is still unclear. In this study, ΔFosB was specifically downregulated by delivering AAV-shRNA into the hippocampus of chronically epileptic mice using the drug-resistant pilocarpine model of mesial temporal epilepsy (mTLE). Immunohistochemistry analyses showed that prolonged downregulation of ΔFosB led to exacerbation of neuroinflammatory markers of astrogliosis and microgliosis, loss of mossy fibers, and hippocampal granule cell dispersion. Furthermore, prolonged inhibition of ΔFosB using a ΔJunD construct to block ΔFosB signaling in a mouse model of Alzheimer’s disease, that exhibits spontaneous recurrent seizures, led to similar findings, with increased neuroinflammation and decreased NPY expression in mossy fibers. Together, these data suggest that seizure-induced ΔFosB, regardless of seizure-etiology, is part of a homeostatic mechanism that protects the epileptic brain from further deterioration.

## Introduction

Epilepsy is a chronic disease affecting more than 70 million individuals worldwide. It is characterized by recurrent unprovoked epileptic seizures (Fisher et al., 2014) that may lead to neurobiological, cognitive, psychological, and social impairments (Fisher et al., 2017; GBD 2016 Neurology Collaborators, 2019). One third of epilepsy patients do not respond to current treatments (symptomatic) (Loscher and Schmidt, 2011), and therefore the need to identify novel therapies that can reverse underlying pathophysiology is high.

Neuronal death, neuronal network rewiring, microgliosis, astrogliosis, alterations in oligodendrocyte functions, and other mechanisms could contribute to drug resistance in epilepsy (Staley, 2015; Vezzani et al., 2019; Sharma et al., 2021; Knowles et al., 2022). We hypothesized that targeting transcription factors that regulate the expression of many genes at the same time could be an effective strategy to reverse or attenuate one or more of the pathologies associated with epilepsy and may enable seizure suppression and disease modification. In this study, we focused on the transcription factor ΔFosB, whose expression in the hippocampus is robustly increased after seizures, associated with cognitive deficits in mouse models of epilepsy or Alzheimer’s disease (AD) neuropathology, and has a long half-life *in vivo* on the order of days, putting it in prime position to exert long-lasting effects on gene expression. We hypothesized that modifying the activity of ΔFosB could have a broad impact on the seizing brain. The ΔFosB protein is encoded by an alternatively spliced variant of the FosB gene, which belongs to Fos protein family of transcription factors. Fos proteins are also called immediate early genes based on their rapid induction in a cell type specific manner including neurons in brain (Choi, 2017). Unlike other Fos proteins, ΔFosB has unusually long half-life that allows it to accumulate and remain in chronically active cells for weeks (Hope et al., 1994; Chen et al., 1997; McClung and Nestler, 2003). Accumulation of ΔFosB has been observed in mouse models of medial temporal lobe epilepsy (mTLE) and in mouse models of AD neuropathology that exhibit spontaneous recurrent seizures (SRSs) (Corbett et al., 2017; You et al., 2017; Stephens et al., 2020). Notably, ΔFosB regulates expression of many genes (McClung and Nestler, 2003; Robison et al., 2013; Lardner et al., 2021) involved in epilepsy-relevant pathways (e.g., excitability and neurotransmission; cellular stress and immunity) (You et al., 2018; Stephens et al., 2020). In addition, ΔFosB activity drives seizure-related cognitive deficits (Eagle et al., 2015; You et al., 2017), one of the major comorbidities of drug-resistant epilepsy (Hermann and Seidenberg, 2007). This combination of unique features (unusually long half-life and epigenetic regulation of many genes) indicates that ΔFosB could serve as a molecular switch that could modify one or more pathological mechanisms associated with epilepsy. Blockade of ΔFosB signaling for several weeks improves cognition in a mouse model of AD neuropathology (Corbett et al., 2017; You et al., 2017); however, whether prolonged blockade of ΔFosB signaling can provide sustained improvement or impact other pathological effects of SRSs is not clear. To address this question, we downregulated ΔFosB expression to inhibit its function in mouse models of mTLE or AD neuropathology over several months. We found that the reduction of ΔFosB signaling for several months exacerbated neuroinflammatory markers and abolished the neuroprotective alterations typically observed in conditions of chronic seizures. These results reveal that ΔFosB plays critical roles in homeostatic mechanisms that protect the epileptic brain from further deterioration.

## Materials and methods

### Adeno-associated viral vector production

Adeno-associated viruses (AAV) were obtained from Vector Biolabs (USA): (1) AAV9-eGFP-U6-m-deltaFosB-shRNA (5′-CACC-GCTGGCCGAGTGAAGTTCAAGT-CTCGAG-ACTTGA ACTTCACTCGGCCAG-TTTTT-3′), (2) AAV9-sGFP-U6-Scrmb- shRNA, or were AAV2 constructs that were previously characterized (Zachariou et al., 2006; Robison and Nestler, 2011) and packaged by the University of North Carolina Vector Core: (3) AAV2-CMV-eGFP, or (4) AAV2-CMV-ΔJunD-IRES2-eGFP that acts as an inhibitor of ΔFosB transcriptional activity by binding and preventing dimerization with other AP-1 factors (Brown et al., 1996). These AAV2 constructs are stably expressed throughout the dentate gyrus within 18–22 days of infusion (Corbett et al., 2017; You et al., 2017).

### Animals

Male C57Bl/6 mice (Janvier, France) 15 weeks of age were used in development/validation of AAV9-GFP-U6-m-deltaFosB-shRNA. These experiments were performed at SynapCell and approved by ethical committee of the High Technology Animal Platform (University Grenoble Alpes, France). Animals were housed in cages on wood litter with free access to food and water until surgery. The animal house was maintained under artificial lighting between 7:30 a.m. to 7:30 p.m. in a controlled ambient temperature (22 ± 2°C) and relative humidity. Male NMRI mice (Charles River, France) weighing 28–32 g (5–6 weeks old) were used in the pilocarpine model of mTLE. Mice were housed in a room with controlled environment (temperature 22 ± 2°C, humidity 55 ± 15%, day/night cycle 12/12 h, light on at 6 a.m.) with food and water *ad libitum*. Experiments were conducted in compliance with guidelines issued by the ethic committee for animal experimentation according to Belgian law and in accordance with the European Committee Council directive (2010/63/EU).

For studies involving mouse models of AD neuropathology, we used heterozygous transgenic mice that express human amyloid precursor protein (APP) carrying Swedish (K670N, M671L) and Indiana (V717F) mutations under control of PDGF-β promoter (Line J20; hAPP770) (Mucke et al., 2000). Littermate controls included age- and sex-matched non-transgenic (NTG) mice. Mice were maintained with 12:12 light/dark cycle in cages with corncob bedding and EnviroPak nesting material, with *ad libitum* access to water and LabDiet 5V5R chow. Mice were group-housed 4–5/cage until appropriate ages for studies, and then were singly housed in a quiet environment for 2 days prior to experimentation and/or sacrifice. APP and NTG mice were studied between the ages of 2 and 5.5-months old. This line of APP mice was chosen for these studies because it has been well-characterized for the spontaneous seizures that they exhibit, and for the relationship between seizures and memory deficits (Palop et al., 2007; Sanchez-Varo et al., 2012; Verret et al., 2012; Corbett et al., 2017). We previously demonstrated that in these APP mice, seizures increase ΔFosB accumulation in the dentate gyrus of the hippocampus, where it epigenetically regulates target genes (Corbett et al., 2017; You et al., 2017, 2018; Stephens et al., 2020), making it ideal for these studies. All experiments were performed in accordance with protocols approved by the Institutional Animal Care and Use Committee of Baylor College of Medicine.

### Development of AAV9-GFP-U6-m-deltaFosB-shRNA in naïve mice

Animals received bilateral injection of AAV (total amount of injected AAV particles; low: 1.7E9; or high: 1.7E10) in both hippocampi. AAV9-eGFP-U6-m-deltaFosB-shRNA was injected to the right side and AAV9-eGFP-U6-Scrmb-shRNA was injected to the left side. Mice were anesthetized with isoflurane, placed into a stereotaxic frame and two small holes were bilaterally opened in the skull. A Hamilton syringe was filled with either vehicle (sterile 1x PBS solution with 5% glycerol) or AAV and the needle inserted into the dorsal hippocampus (coordinates from bregma; anteroposterior: −2 mm; mediolateral: ± 1.75 mm; dorsoventral: −2.1 mm). One μl of vector per hemisphere was injected at a speed of 0.10 μl/min. Tissues were collected 4 weeks after AAV injection. Mice were deeply anesthetized with isoflurane and perfused with 1x PBS solution containing heparin, and brains were rapidly removed. Both hippocampi were resected and stored at −80°C.

### Mouse pilocarpine model of mTLE

NMRI mice were intraperitoneally (i.p.) injected with 300 mg/kg of pilocarpine (Sigma-Aldrich), as previously described (Mazzuferi et al., 2012). *N*-methylscopolamine bromide (Sigma-Aldrich, 1 mg/kg, i.p.) was administered 30 min prior to pilocarpine injection to limit peripheral cholinergic effects of pilocarpine. Status epilepticus (SE) typically appeared within the first hour after pilocarpine injection and was characterized by continuous stage 3–5 seizures (continuous tonic-clonic seizures) scored according to the Racine’s scale (Racine, 1972) during at least 30 min. Diazepam (10 mg/kg; Roche S.A, Brussels, Belgium) was administered i.p. 3 h after SE onset to reduce the duration of SE. After SE, all animals were intraperitoneally injected with lactated Ringer solution and fed with soaked rodent food. Age-matched and gender-matched control NMRI mice (sham group) were injected i.p. with *N*-methylscopolamine, diazepam and lactated Ringer but received saline instead of pilocarpine.

Mice surviving SE typically showed spontaneous recurrent seizures (SRSs) within days to weeks after SE induction by pilocarpine injection (Mazzuferi et al., 2012). Seizures were monitored using simultaneously video recording and monitoring of locomotor activity with a 3D micro-accelerometer ship secured to the mouse, as previously described (Srivastava et al., 2018). Only secondary generalized seizures Racine’s score 3–5 (Racine, 1972) were detected, reviewed and confirmed manually with video by an experienced experimenter blinded to treatment. Seizure duration, severity and frequency were quantified. All mice included in the present study entered SE after pilocarpine injection and developed stage 3–5 SRSs, which were confirmed with video-accelerometry before injecting the virus (see Supplementary Figures 2A–C).

### Stereotaxic virus injection

Seven weeks after SE induction, SE mice and control sham mice were anesthetized with 2–3% isoflurane (Oxygen: 1.5 L/min), placed into a stereotaxic frame and two small holes were bilaterally opened in the skull. A Hamilton syringe was filled with either vehicle (sterile 1x PBS solution with 5% glycerol) or AAV (1.7E13 viral particles per ml prepared in 1x PBS solution with 5% glycerol) and the needle inserted into the dorsal hippocampus (coordinates from bregma; anteroposterior: −2 mm; mediolateral: ± 1.75 mm; dorsoventral: −2.1 mm). One μl of vector per hemisphere was injected at a speed of 0.10 μl/min using a microsyringe pump controller. Mice were allowed to recover for 7 days before monitoring seizures with video-accelerometry. All SE mice were monitored with video-accelerometer to quantify the number of SRSs over 14 days before AAV injection. SE mice with comparable number of SRSs before treatment were randomized to receive vehicle, AAV9-eGFP-U6-Scrmb-shRNA (Neg shRNA) or AAV9-GFP-U6-m-deltaFosB-shRNA (ΔFosB shRNA). Control sham mice received vehicle only. For APP and NTG mice (anesthesia was induced by 2–3% isoflurane and maintained using 1–1.5% isoflurane; Oxygen: 1.5 L/min), bilateral DG targeting was achieved by stereotaxic infusion of 1 μl of titer-matched (≤5 × 10^12^) AAV2 solution into the dentate gyrus at rostral (coordinates from bregma; anteroposterior: −1.7 mm; mediolateral: 1.2 mm; dorsoventral: 2 mm) and caudal (coordinates from bregma; anteroposterior: −2.7 mm; mediolateral: 2 mm; dorsoventral: 2.1 mm) coordinates. Mice were allowed to express AAVs for either 4 or 12 weeks, until experimentation and/or euthanasia and brain collection. Virus expression was confirmed using immunohistochemical detection of eGFP or ΔJunD.

### Tissue sampling

Control sham mice and SE mice were deeply anesthetized with 2–3% isoflurane (Oxygen: 1.5 L/min), either 4 or 8 weeks after vehicle or AAV brain injection. They were perfused sequentially via the left ventricle with 30 ml chilled 1x PBS solution containing 10IU/ml heparin, and brains were rapidly removed. The left-brain hemisphere was immersed in 4% paraformaldehyde (PFA) in PBS solution (pH7.4) for 3 h at room temperature, and then soaked in a 15% sucrose solution and stored at 4°C. The dorsal hippocampus was rapidly extracted from the right brain hemisphere, snap frozen in liquid nitrogen, and then stored at −80°C until processing for western blotting and qPCR analyses.

APP and NTG mice were deeply anesthetized using SomnaSol Euthanasia-III solution (390 mg pentobarbital sodium and 50 mg phenytoin sodium; Henry Schein) prior to transcardial perfusion with chilled saline and rapid removal of brains. The right hemibrain was drop-fixed in 4% PFA in PBS solution for 48 h, and then rinsed in PBS for 24 h prior to cryoprotection in 30% sucrose and stored at 4°C. The left hemibrain was snap frozen on dry ice and stored at −80°C until isolation of the hippocampus and processing for qPCR analysis.

### RNA extraction and qPCR

RNA was extracted from the dorsal hippocampus 4 weeks after AAV injection using RNeasy minikit (Qiagen 74134). A total of 500 ng of RNA was reverse transcribed by high-capacity cDNA RT Kit + RNase Inhibitor (Applied Biosystems cat no 4374966). qPCR was performed using Universal Master Mix (Life Technologies ref 43004437) on CFX384 (BioRad). TaqMan probes (ThermoFisher) were used to detect the gene expression [endogenous controls: Bcl2l13 (Mm00463355_m1)], Brap (Mm00518493_m1); ΔFosB (ARXGTN9); FosB (ARU64JE) and ΔΔCt method was used to determine differential expression.

### Protein extraction and western blotting

Total proteins were extracted from dorsal hippocampus at 4 and 8 weeks after AAV injection using 2X #9803 Cell Lysis Buffer (Cell signaling). Twenty micrograms of total proteins were loaded per well of SDS-PageNovex 8–16% gel, then transferred to a PVDF membrane (Millipore). The membrane was blocked in Odyssey blocking buffer (LI-COR), and ΔFosB was detected using ΔFosB antibody (Cell Signaling #14695S; dilution 1:2000). Actin B was used as loading control (LI-COR; dilution 1:10000). Secondary antibody (LI-COR) was diluted 1:5000. Images were acquired with a LI-COR CLX and visualized using Image Studio.

### Tissue processing and immunohistochemistry

For mTLE model mice and controls, sectioning of the mouse brain hemispheres was performed by Neuroscience Associates (Knoxville, TN, USA) and immunohistochemistry was carried out by indirect immunofluorescence. The list of antibodies used in this study is provided in Supplementary Table 1. Free-floating coronal sections (40 μm-thick) were obtained using a cryostat microtome and permeabilized 15 min in Tris-buffered saline (TBS) containing 0.3% Triton X-100 (TBS-T). Then, sections were incubated overnight at room temperature with the primary antibody diluted in TBS-T. After three washes of 5 min in TBS, they were incubated for 1 h at room temperature with secondary antibody and 4′,6-diamidino-2-phenylindole (DAPI, 300 nM) prepared in TBS-T, rinsed 3 times 5 min in TBS, and finally mounted on glass slides using Fluoromount mounting medium (Thermo Fisher Scientific).

For APP and NTG mice, tissue preparation and immunohistochemistry were performed as previously described (Corbett et al., 2017; You et al., 2017; Stephens et al., 2020). Hemibrains were sectioned at 30 μm into ten coronal subseries throughout the rostral-caudal extent of the brain using a freezing, sliding microtome. Sections were stored in cryoprotectant medium (30% glycerol, 30% ethylene glycol, 40% PBS). 3′3-diaminobenzidine (DAB; Sigma-Aldrich) immunolabeling of NPY, Iba1, and GFAP was performed using the primary and biotinylated secondary antibodies indicated in Supplementary Table 1.

### Image acquisition and analysis

For mTLE model mice and controls, whole slide imaging was performed using an Axioscan Z1 scanner (Zeiss) with 20x objective. For each experiment, digital acquisitions were performed using consistent exposure parameters, avoiding overexposure, to ensure accurate signal quantification between conditions. Image analysis was performed with VisioPharm 6 software (VisioPharm, Hørsholm, Denmark) as described previously (Albert et al., 2019). Regions of Interest (ROI), such as the whole hippocampus or granular layer of the dentate gyrus, were delineated manually, and automatic quantification of the immunoreactive signal was performed using a linear Bayesian algorithm, providing a value of signal area (marker area in μm^2^). Then, a % marker coverage was calculated (i.e., ratio between the immunoreactivity signal area in μm^2^ and the area of the ROI in μm^2^). In specific cases, number of cells and cell density (number of cells per tissue area) were quantified using Visiopharm. Percent marker coverage, cell number and cell density were quantified in the dorsal and horizontal part of the hippocampus (Bregma −1.34 to −2.54 mm) on 3 to 4 sections per animal using well-defined landmarks based on a mouse brain atlas (Paxinos and Franklin, 2019). All quantifications were done blindly until the end of statistical analysis.

For APP and NTG mice, brightfield microscope images (Zeiss) of coronal brain sections immunostained using DAB as the chromagen were quantified and analyzed using ImageJ. Iba1 and GFAP signal intensity were quantified using the Measure function to calculate the % Area that contained signal intensity above a consistent pre-set threshold within the bounds of the dentate gyrus, averaged from 2 rostral coronal sections. NPY was quantified using the Measure function of ImageJ to measure the “mean gray value” (signal intensity average within a traced ROI) of DAB signal present in the region of mossy fiber tracts projecting to CA3 that are well-known to exhibit robust ectopic NPY expression in mouse models of mTLE and AD. The mean gray values were measured from the mossy fiber ROI and in an adjacent area of similar shape/size in the stratum radiatum immediately superior to the ROI. The average signal measured in the mossy fiber ROI in 2 coronal sections was then divided by the average signal measured in the stratum radiatum of the same sections, and expressed as a fold change relative to NTG-GFP mice.

### ChIP-sequencing and gene ontology network analysis

Chromatin immunoprecipitation and sequencing (ChIP-seq) was performed in samples of whole hippocampus harvested and processed from 2 to 4-month old NTG and APP mice as described in You et al. (2018) and Stephens et al. (2020). The Cytoscape (v3.9.1) platform was used to perform new ClueGO (v2.5.9) gene ontology (GO) analyses (Shannon et al., 2003; Bindea et al., 2009) on the respective sets of all target genes found to be significantly bound by ΔFosB in our published hippocampal ΔFosB ChIP-seq analyses (Stephens et al., 2020) in pilocarpine, vehicle, non-transgenic and APP mice or the set of 442 ΔFosB target genes shared by Pilo and APP mice and not respective controls. Using ClueGO, a two-tailed hypergeometric test with a Benjamini-Hochberg correction (Benjamini and Hochberg, 1995) was used to calculate significant enrichment of Biological Process GO Terms (ontology version: 5/25/2022) with the respective sets of ΔFosB target genes in pilocarpine, vehicle, non-transgenic and APP mice. ClueGO was also used to generate a graphical GO Network from the 442 ΔFosB target genes shared by pilocarpine and APP mice in which GO Terms enriched with ΔFosB target genes are displayed as functionally grouped nodes. GO Term nodes are connected by lines (edges) indicating that target genes are shared between nodes, and node size increases as a function of GO Term enrichment significance. Once generated from given parameters, GO analyses and networks have been simplified to remove redundant and non-brain organ-specific GO Terms and filtered to only include GO Terms that are related to processes of immunity and/or neuroprotection. GO Network parameters that were changed from ClueGO default settings are as follows: FDR < 0.05 (Figures 6A, B) or 0.5 (Figure 6C), GO Level range = 3–20, minimum number of genes in GO Term = 1, minimum percentage of genes in GO Term = 0.1%, kappa = 0.62 (Figures 6A, B) or 0.67 (Figure 6C), and GO Term fusion = TRUE.

### Statistical analysis

GraphPad Prism 9.2.0 software was used to perform all statistical analysis. To determine differences between groups the one-way or two-way ANOVA or the non-parametric Kruskal-Wallis were applied as appropriate. Follow up pairwise comparisons were done using Tukey’s *post-hoc* test or Two-stage linear step-up procedure of Benjamini, Krieger and Yekutieli or Benjamini-Hochberg FDR *post-hoc* testing as appropriate. Proportions were compared as appropriate with the Chi-square test. The level of significance was set at *p* < 0.05. Data are presented as mean ± SEM.

## Results

### Development of a new specific inhibitor of ΔFosB

ΔFosB, JunD, and other proteins of the Fos, Jun, ATF, and Maf subfamilies are members of the activator protein-1 (AP-1) complex of transcription factors (Wu et al., 2021) that have a critical function in a wide range of tissues and pathways. A number of studies have demonstrated that ΔFosB plays critical role in epigenetic regulation of gene expression in the brain under physiological conditions (Eagle et al., 2018) and when neuronal activity is chronically stimulated, such as in the nucleus accumbens after drugs of abuse (Robison and Nestler, 2011), and in the dentate gyrus (DG) of the hippocampus in conditions with recurrent seizures (Corbett et al., 2017; You et al., 2017; Stephens et al., 2020). The use of the experimental construct ΔJunD (dominant negative mutant variant of JunD) to block downstream epigenetic actions of ΔFosB (Winstanley et al., 2007) has been instrumental in deciphering the role of ΔFosB in the regulation of neuronal functions, particularly in conditions of chronic activity that lead to accumulation of ΔFosB. However, it is still possible that some of the observed effects are due to binding of ΔJunD to other AP-1 complex members. We therefore developed a shRNA specifically targeting the mRNA of ΔFosB and not its parent transcript FosB (Supplementary Figure 1A). To confirm specificity of the newly developed shRNA, AAV-ΔFosB-shRNA was injected into dorsal hippocampus of healthy mice and qPCR (with specific primers for ΔFosB and FosB transcripts) was performed 4 weeks later. We showed that ΔFosB-shRNA can specifically downregulate ΔFosB mRNA in a dose-dependent manner without any effect on FosB mRNA expression (Supplementary Figures 1B, C). To further strengthen value of newly developed AAV-ΔFosB-shRNA as specific ΔFosB inhibitor, we assessed if c-Fos protein expression [previously confirmed ΔFosB downstream target (Corbett et al., 2017)] changed when ΔFosB protein is downregulated in mTLE mouse model (described later). We confirmed that subtle decrease of ΔFosB at 4 weeks led to an increase in c-Fos expression in the hippocampus and DG (Supplementary Figures 3B, C). Similarly, we observed robust increase in c-Fos protein expression at 8 weeks upon AAV injection (Supplementary Figure 3B).

### Administration of an AAV-ΔFosB shRNA led to sustained downregulation of ΔFosB protein in the hippocampus and marked modification of the hippocampal cytoarchitecture of mTLE mouse model

**FIGURE 1:**
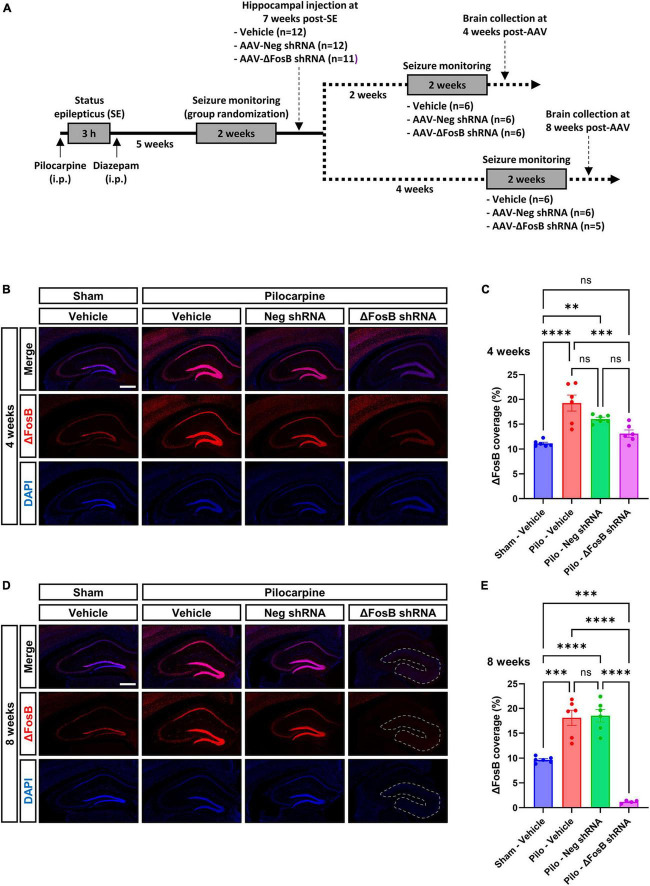
Experimental design and validation of ΔFosB knockdown. **(A)** Schematic diagrams showing the experimental design used in the study. Pilocarpine was administered to induce SE followed weeks later by the development of SRSs. SRSs were monitored before AAV administration and SE mice with comparable number of SRSs were randomized to receive vehicle, AAV-Neg shRNA or AAV-ΔFosB shRNA. SRSs were monitored again for 2 weeks starting at 2 weeks or 4 weeks after AAV injection. Terminal collection of the brains was performed at the end of each seizure monitoring phase at 4 weeks or 8 weeks after AAV injection. **(B–E)** Representative hippocampal immunohistochemistry images and quantification of ΔFosB expression levels in the dorsal part of the hippocampus at **(B,C)** 4 weeks and **(D,E)** 8 weeks after vehicle or AAV delivery. 4′,6-diamidino-2-phenylindole (DAPI) was used as a nuclear counterstain. Note that the morphology of the granular layer of the dentate gyrus is markedly affected at 8 weeks with the ΔFosB shRNA, presenting a general size enlargement and decreased DAPI signal intensity (area annotated in D). Data are expressed as mean with standard error of the mean (SEM). Statistical tests: ANOVA followed by Tukey’s *post-hoc* test (**p < 0.01; ***p < 0.001; ****p < 0.0001; ns: non-significant). Scale bars = 500 μm.

To assess the effects of ΔFosB suppression in the brains of mice in a therapeutically relevant timeframe, AAVs expressing ΔFosB-specific shRNA or negative control (neg) were injected bilaterally in the dorsal hippocampus of chronically epileptic mice displaying spontaneous recurrent seizures (SRSs) 7 weeks after status epilepticus (SE) induced by pilocarpine injection (Experimental design in Figure 1A; Seizures in Supplementary Figures 2A, B). Due to unusually long half-life of the ΔFosB protein (Hope et al., 1994; Chen et al., 1997; McClung and Nestler, 2003), we assessed its downregulation 4 and 8 weeks after AAV injection by immunohistochemistry (Figures 1B–E) and western blotting (Supplementary Figure 2D) in the dorsal hippocampus. In vehicle-treated sham animals, ΔFosB was localized in pyramidal neurons of the Ammon’s Horn (CA1 to CA3) and granule cells of the DG (Figures 1B, D). In vehicle-treated mTLE mice, ΔFosB immunoreactive signal increased significantly, especially in neurons of the CA1 and granule cells of the DG at 4 and 8 weeks (Figures 1B–E). Administration of the AAV-Neg shRNA had no impact on ΔFosB levels in mTLE mice at 4 weeks (Figures 1B, C) or 8 weeks (Figures 1D, E). In contrast, AAV-ΔFosB shRNA injection resulted in a moderate but significant downregulation of ΔFosB at 4 weeks, comparable to physiological levels (sham animals injected with vehicle, Figures 1B, C). Strikingly, ΔFosB shRNA led to an almost complete disappearance of the ΔFosB signal in the whole dorsal hippocampus of mTLE mice at 8 weeks, along with a marked modification of the hippocampal cytoarchitecture based on DAPI staining (Figures 1D, E). Whereas the CA1 layer of the hippocampus and the granule cell layer of the DG were clearly demarcated in mTLE mice injected with vehicle or AAV-Neg shRNA, these structures were no longer obviously defined at 8 weeks in mTLE mice injected with AAV-ΔFosB shRNA (Figure 1D).

To determine whether the downregulation of ΔFosB protein could have an additional impact on the seizure development, epileptic mice were continuously monitored for seizure detection for 14 days beginning at 2 and 6 weeks after AAV injection. The frequency, duration and severity of SRSs remained similar after ΔFosB protein downregulation, suggesting that there was no influence of ΔFosB protein downregulation on seizure development (Supplementary Figures 2A–C). It is noteworthy to mention that AAV-ΔFosB shRNA was delivered to the dorsal hippocampus only. TLE is characterized by localization of seizure foci in multiple brain areas including the hippocampus, entorhinal cortex, or amygdala (Bartolomei et al., 2005). Thus, the lack of effect on seizures may be explained by the limited brain coverage we achieved here with the genetic tool (restricted to the dorsal hippocampus), remaining not sufficient to counteract the occurrence of seizures originating from different brain regions. Another explanation could be the low sample size in each group for seizure monitoring (*n* = 5–6; each group), leading to a statistically underpowered study to capture differences in seizure parameters.

Overall, these observations indicated correct targeting of the dorsal hippocampus with AAVs, and efficient and selective downregulation of ΔFosB protein with the shRNA 8 weeks after viral delivery with concomitant change in hippocampal morphology in mTLE mice.

### Downregulation of ΔFosB induced granule cell dispersion (GCD) in the hippocampus of mTLE mice

**FIGURE 2:**
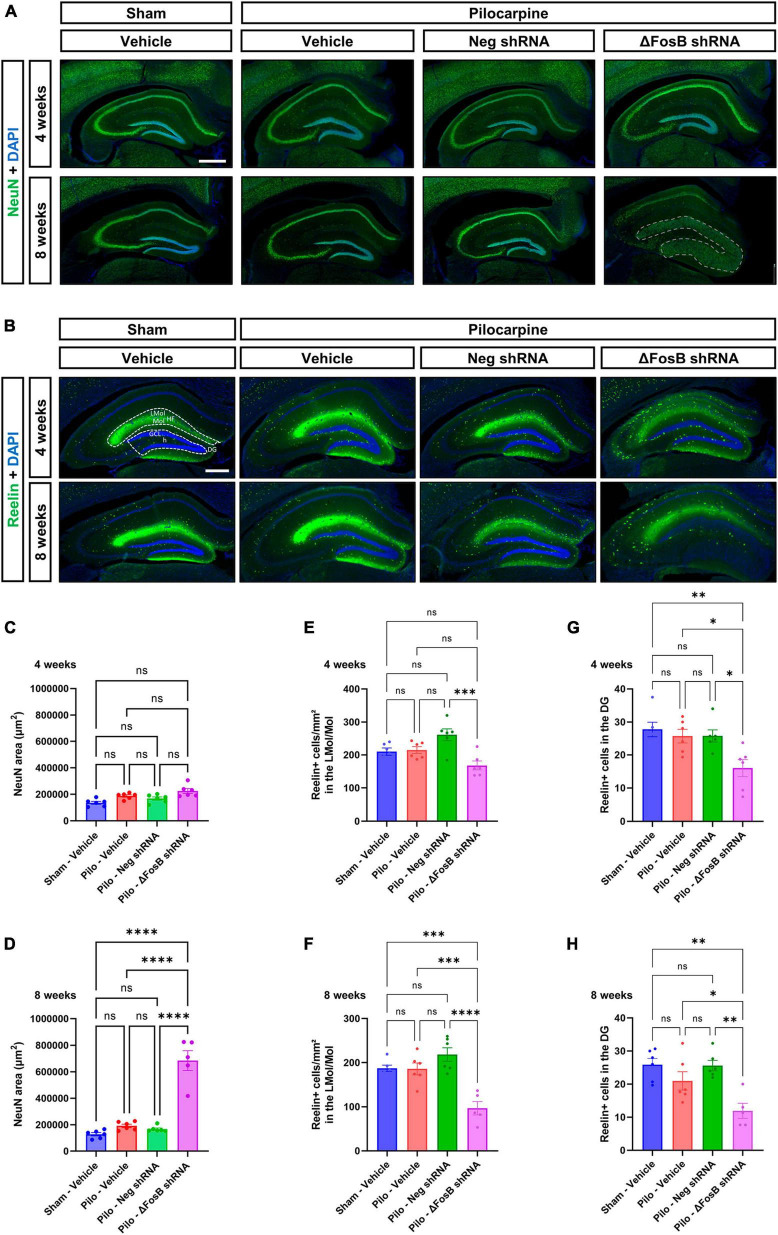
ΔFosB downregulation leads to granular cell dispersion and disruption of the Reelin signaling pathway in the dentate gyrus of the mTLE mouse model. **(A)** The neuronal marker NeuN was used to visualize the morphology and cytoarchitecture of the hippocampus. Note that the morphology of the granular layer of the dentate gyrus was markedly affected at 8 weeks with the ΔFosB shRNA [area annotated in **(A)**]. The area of the granular layer was measured at **(C)** 4 weeks and **(D)** 8 weeks. **(B)** The density of Reelin + cells was analyzed by immunohistochemistry in the dorsal part of the hippocampus, **(E,F)** specifically in the area surrounding the hippocampal fissure (HF) [annotated in **(B)**] including the lacunosum moleculare layer of the hippocampus (LMol) and the upper third of the molecular layer of the dentate gyrus (Mol) at 4 weeks and 8 weeks after vehicle or AAV delivery. **(G,H)** In addition, the number of Reelin + cells was quantified in the granule cell layer (GCL) of the dentate gyrus (DG) [including the hilus (h) of the dentate gyrus; annotated in **(B)**]. Data are expressed as mean with standard error of the mean (SEM). Statistical tests: ANOVA followed by Tukey’s *post-hoc* test (*p < 0.05; **p < 0.01; ***p < 0.001; ****p < 0.0001; ns: non-significant). Scale bars = 500 μm.

We observed that ΔFosB downregulation led to significant histopathological alterations in the hippocampus of mTLE mice, as evidenced by DAPI staining (Figure 1D). To further characterize these pathological findings, we analyzed the neuronal architecture using immunohistochemistry with the neuronal marker NeuN. No difference in neuronal cytoarchitecture was observed in the hippocampus of mTLE mice treated with vehicle or AAV-Neg shRNA compared to sham animals treated with vehicle (Figure 2A). Furthermore, while ΔFosB knockdown did not alter the hippocampal morphology at 4 weeks (Figure 2A; granule cell area measured in Figure 2C), significant GCD was observed at 8 weeks as shown by the spread of NeuN staining (Figure 2A; granule cell area measured in Figure 2D). The granule cell phenotype of the dispersed cells at 8 weeks was corroborated by immunohistochemistry for Prox1, a marker specific to granular cells of the DG (Supplementary Figure 3A).

One of the potential mechanisms contributing to the observed GCD in the pilocarpine mouse model involves the disruption of Reelin signaling which plays a pivotal role in modulating neuronal migration and positioning during brain development (Hirota and Nakajima, 2017). In order to explore this hypothesis, we conducted a comprehensive analysis of Reelin expression within the hippocampus utilizing immunohistochemistry. Noteworthy reduction in Reelin protein levels, particularly evident within an area that includes both the lacunosum moleculare layer of the hippocampus and the upper third of the molecular layer of the DG (Figures 2B–F), and in the granular layer of the dentate gyrus (Figures 2B–H) was observed. This decline was observed at 4 and 8 weeks following the administration of AAV-mediated ΔFosB knockdown.

Our observations suggest that ΔFosB may maintain Reelin signaling and thereby help preserve the positioning of granule cells in conditions with seizure activity.

### Downregulation or inhibition of ΔFosB reverses “protective” adaptations of mossy fibers in the hippocampus of mice with recurrent seizures

**FIGURE 3:**
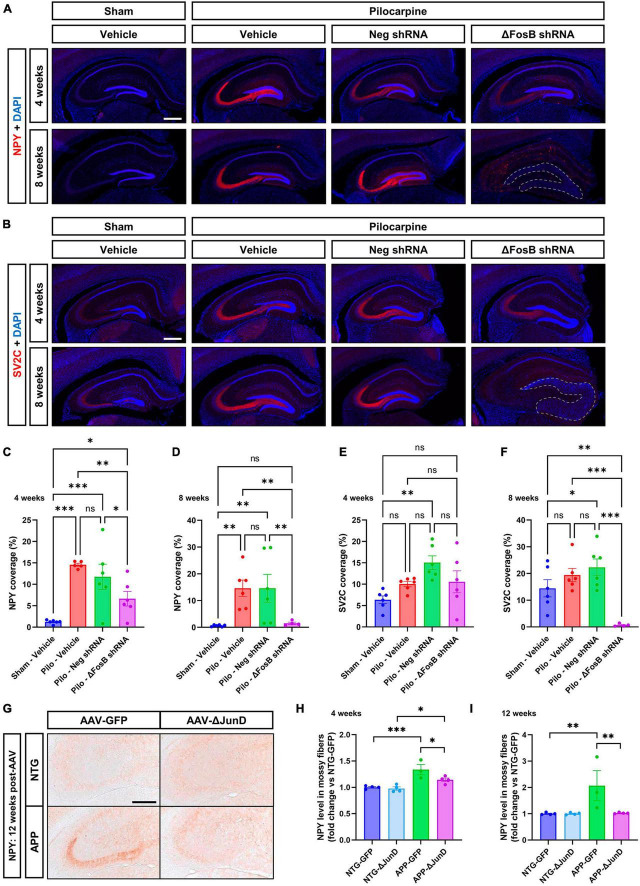
ΔFosB inhibition reduces expression of NPY and SV2C in mossy fibers in epileptic brain. **(A)** NPY (neuropeptide Y) and **(B)** SV2C (synaptic vesicle glycoprotein 2C) were used as markers of mossy fibers in the hippocampus. NPY and SV2C immunoreactive signal was quantified in the dorsal part of the hippocampus at **(C,E)** 4 weeks and **(D,F)** 8 weeks after vehicle or AAV delivery. Data are expressed as mean with standard error of the mean (SEM). **(G)** Representative immunostaining of NPY in the dentate gyrus of NTG or APP mice that expressed AAV-GFP or AAV-ΔJunD for 12 weeks. Quantification of NPY expression after **(H)** 4 weeks or **(I)** 12 weeks of AAV expression. Statistical tests: Two-factor ANOVA followed by Two-stage linear step-up procedure of Benjamini, Krieger and Yekutieli **(A–F)** or Benjamini-Hochberg FDR (H-I) *post hoc* testing (*p < 0.05; **p < 0.01; ***p < 0.001; ns: non-significant). Scale bars in **(A,B)** = 500 μm or in **(G)** = 250 μm.

It was shown that application of Reelin in older mice or in a model of a neurodevelopmental disorder improves synaptic function (↑ dendrite spine density and LTP) (Rogers et al., 2011) or restores behavioral and morphological deficits (mossy fibers; MF) (Ibi et al., 2020) in the hippocampus. To determine the impact of ΔFosB downregulation on MF we used two axonal markers, NPY (neuropeptide Y) and SV2C (synaptic vesicle glycoprotein 2C), that are typically increased in expression in MF during abnormal structural reorganization of the DG in brains of mTLE patients (Crevecoeur et al., 2014) and animal models (Nadler et al., 2007; Srivastava et al., 2018). In the mTLE mouse model, we confirmed the increase in NPY (Figures 3A–D) and SV2C (Figures 3B–F) in the MF tracts in the dorsal hippocampus of pilocarpine mice treated with vehicle or AAV-Neg shRNA, compared to sham animals treated with vehicle. Downregulation of ΔFosB reduced NPY at 4 weeks (Figures 3A–C, E) and caused expression of both NPY and SV2C to be nearly absent at 8 weeks (Figures 3A, B, D, F).

To determine if ΔFosB is required to reverse these MF adaptations across conditions with recurrent seizures regardless of etiology, we performed analogous experiments in which we expressed AAV-GFP or AAV-ΔJunD to block ΔFosB activity for 4 or 12 weeks, and measured induction of NPY-positive MF in transgenic human amyloid precursor protein (APP) mice or non-transgenic (NTG) controls. This APP mouse model of AD neuropathology (Line J20) exhibits recurrent epileptiform spikes and seizure activity beginning in the first months of life (Fu et al., 2019). We have reported that epileptiform activity in pilocarpine or APP mice promotes hippocampal accumulation of ΔFosB and downstream alterations in ΔFosB target gene expression that can impair memory (Corbett et al., 2017; You et al., 2017; Fu et al., 2019). These mice also exhibit increased expression of NPY in MF (Palop et al., 2007; Roberson et al., 2011). Notably, overexpression of ΔJunD reduced NPY signal in APP mice 4 weeks after expression and led to nearly complete reduction by 12 weeks after expression (Figures 3G–I). These results suggest that increased expression of ΔFosB is critical for MF adaptive response in the epileptic brain.

### Prolonged downregulation or inhibition of ΔFosB increased neuroinflammation in the hippocampus of seizing mouse brain

**FIGURE 4:**
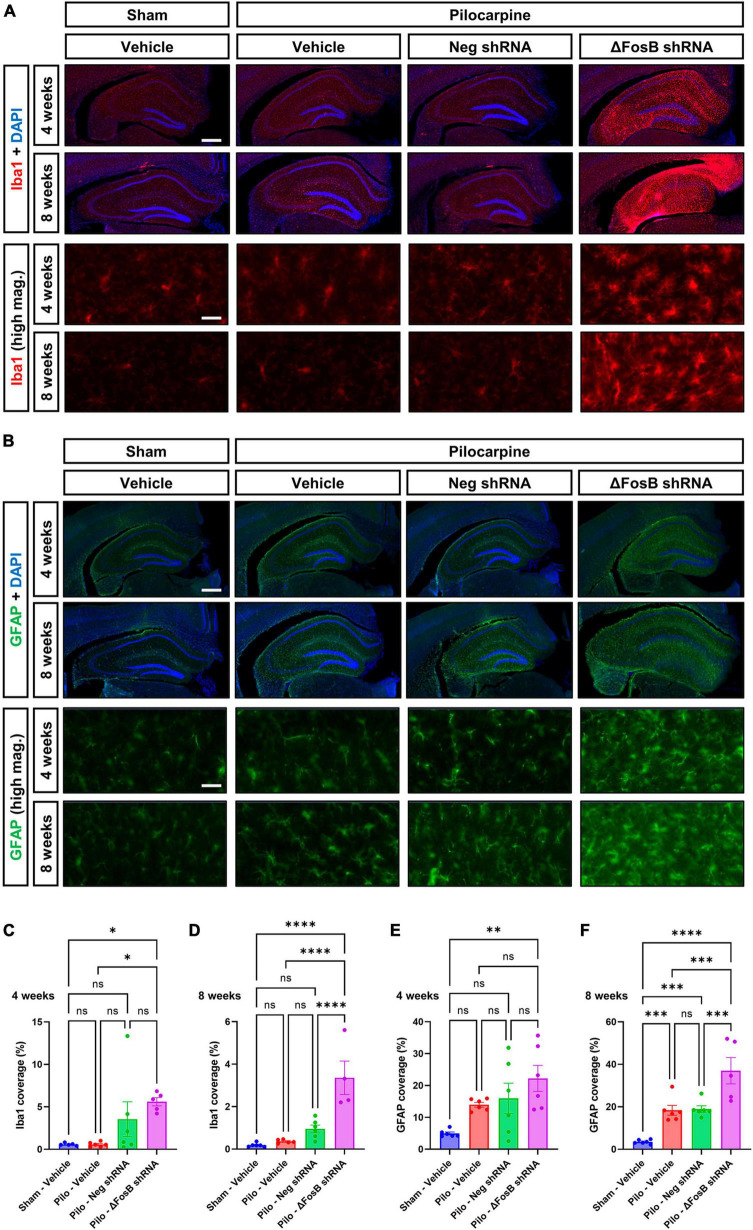
Prolonged ΔFosB inhibition exacerbates neuroinflammation in epileptic brain. **(A)** Iba1 (ionized calcium binding adaptor molecule-1) and **(B)** GFAP (glial fibrillary acidic protein) were used as markers of microglia and astrocytes, respectively. High-magnification images (high mag.) in panels **(A,B)** illustrate the morphology of microglia and astrocytes in the hippocampus under the different experimental conditions. Iba1 and GFAP immunoreactive signal was quantified in the dorsal part of the hippocampus at **(C,E)** 4 weeks and **(D,F)** 8 weeks after vehicle or AAV delivery. Data are expressed as mean with standard error of the mean (SEM). Statistical test: ANOVA followed by Two-stage linear step-up procedure of Benjamini, Krieger and Yekutieli *post-hoc* testing (*p < 0.05; **p < 0.01; ***p < 0.001; ****p < 0.0001; ns: non-significant). Scale bars = 500 μm in low-magnification pictures and 50 μm in high-magnification pictures.

Neuroinflammation is another hallmark of recurrent seizures observed in mTLE or AD patients (Kandratavicius et al., 2015; Kinney et al., 2018), and in respective animal models (Zhu et al., 2017; Srivastava et al., 2018). Due to the marked changes in dorsal hippocampus morphology, we hypothesized that ΔFosB protein inhibition would increase neuroinflammation in the brains of mice with recurrent seizures. Microglial cells and astrocytes were analyzed by immunohistochemistry using Iba1 and GFAP markers, respectively (Becker et al., 2021). Upon vehicle treatment, pilocarpine mice exhibited no significant changes in Iba1 levels at 4 or 8 weeks compared to sham mice (Figures 4A–D), while in contrast, GFAP levels were markedly enhanced in the mTLE mouse model at both time points (Figures 4B–F). Administration of AAV-ΔFosB shRNA in mTLE mice resulted in a marked increase in Iba1 and GFAP levels notably at 8 weeks in the dorsal hippocampus (compared to AAV-Neg shRNA treatment), suggesting that ΔFosB downregulation exacerbated microglial activation and astrogliosis (Figures 4A–F). Further evidence of astrocyte and microglia activation at 4 and 8 weeks following ΔFosB downregulation was suggested by the morphological enlargement of these cells in the hippocampus (High magnification pictures in Figures 4A, B).

**FIGURE 5:**
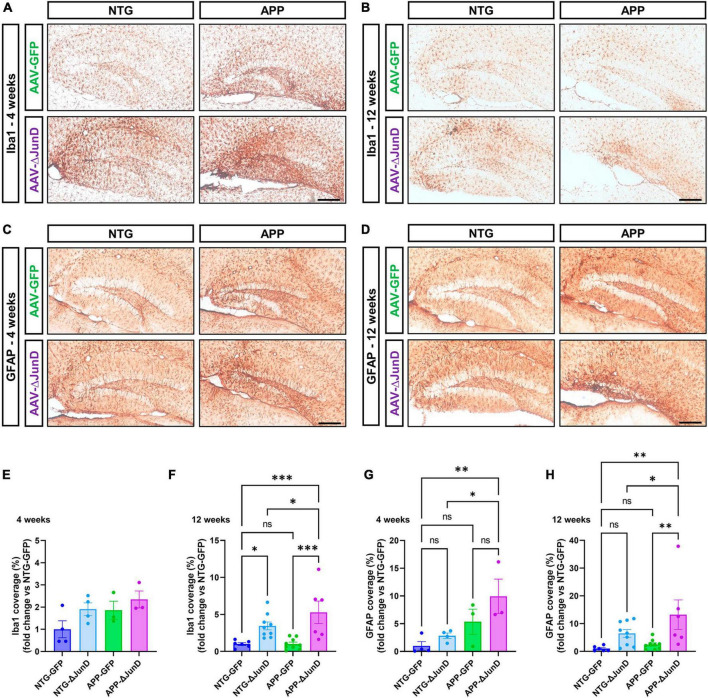
Prolonged ΔFosB inhibition exacerbates neuroinflammation in APP mice. **(A,B)** Iba1 and **(C,D)** GFAP were used as markers of microglia and astrocytes, respectively. Iba1 and GFAP immunoreactive signal was quantified in the dentate gyrus of the hippocampus at **(E,G)** 4 weeks and **(F,H)** 12 weeks after AAV delivery. Data are expressed as mean with standard error of the mean (SEM). Statistical tests: Two-factor ANOVA followed by Benjamini-Hochberg FDR *post-hoc* test (*p < 0.05; **p < 0.01; ***p < 0.001; ns: non-significant). Scale bars = 250 μm.

To determine if ΔFosB is similarly required to suppress immune cell reactivity across conditions with recurrent seizures regardless of etiology, we measured induction of Iba1 and GFAP in APP mice and NTG controls 4 or 12 weeks after infusion with AAV-GFP or AAV-ΔJunD. We found that 4-week expression of AAV-ΔJunD had no effect on Iba1 levels in the dentate gyrus of NTG-ΔJunD or APP-ΔJunD mice compared to respective AAV-GFP controls (Figures 5A, E). However, after 12 weeks of ΔFosB blockade, Iba1 levels were robustly increased in NTG-ΔJunD and APP-ΔJunD mice compared to respective AAV-GFP controls (Figures 5B, F). Iba1 levels were also significantly higher in APP-ΔJunD than NTG-ΔJunD mice after 12-week ΔFosB blockade (Figures 5B, F). GFAP expression did not differ in NTG-ΔJunD or APP-ΔJunD mice compared to respective AAV-GFP controls at 4 weeks post-AAV infusion, although GFAP expression was significantly higher in APP-ΔJunD than NTG-ΔJunD mice after 4-week ΔFosB blockade (Figures 5C, G). Similar to Iba1 levels after 12-week ΔFosB blockade, GFAP levels were robustly increased in NTG-ΔJunD and APP-ΔJunD mice compared to respective controls, and GFAP expression was also higher in APP-ΔJunD than NTG-ΔJunD mice (Figures 5D, H).

These results indicate that ΔFosB regulates similar functions (e.g., MF adaptive response, neuroinflammation) in brains with recurrent seizures irrespective of seizure etiology.

### A subset of hippocampal ΔFosB target genes in pilocarpine or APP mice are involved in immunity and neuroprotection

**FIGURE 6:**
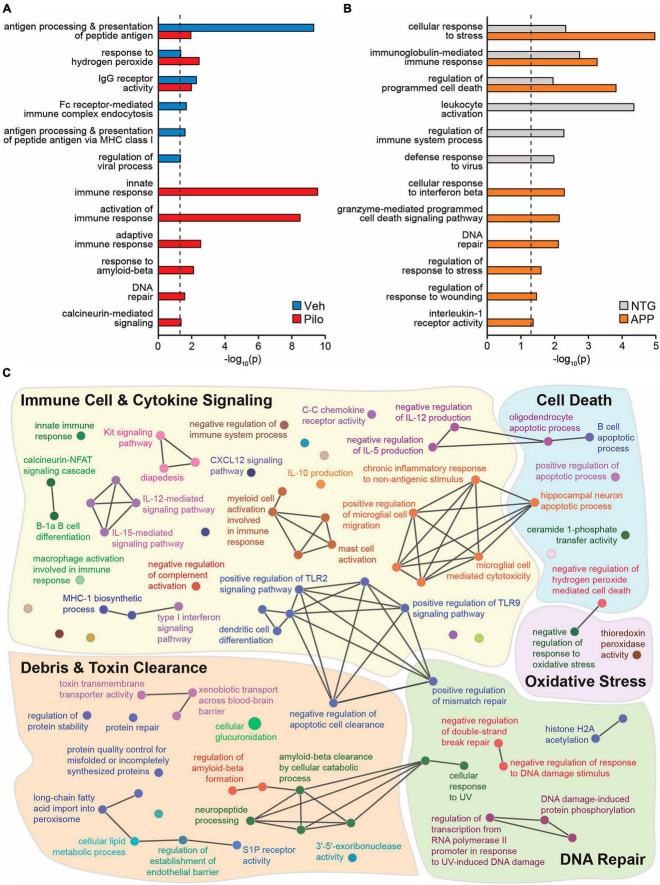
Across mouse models with recurrent seizures but not controls, ΔFosB binds to key target genes in the hippocampus involved in immunity and neuroprotection. Hippocampal ΔFosB target genes (Stephens et al., 2020) functionally enrich GO Biological Process Terms related to immunity and/or neuroprotection in **(A)** wildtype mice injected with Vehicle vs. Pilocarpine or in **(B)** NTG vs. APP mice. Enrichment *p*-values for each highlighted GO Term are depicted on the *x*-axis with a log_10_ scale for ease of comparison. The dashed black line denotes the threshold of significance under two-sided hypergeometric testing with Benjamini-Hochberg FDR = 0.05. **(C)** A GO Biological Process network shows GO Terms related to Immune Cell & Cytokine Signaling (yellow), Cell Death (blue), Oxidative Stress (purple), DNA Repair (green), and Debris & Toxin Clearance (red) that are enriched with the set of 442 hippocampal ΔFosB target genes that are shared between Pilo and APP mice *and not respective control mice*. Nodes indicate individual GO Terms and lines between nodes indicate that connected nodes share genes. Nodes of larger size denote GO Terms enriched at *p* = 0.05 and nodes of smaller size denote GO Terms enriched at *p* = 0.5.

To identify novel putative mechanisms by which ΔFosB activity might suppress immune reactivity and pro-inflammatory signaling across conditions with recurrent seizure activity, we performed new Gene Ontology (GO) Biological Process analyses of hippocampal ΔFosB target genes. Using previously published ChIP-seq datasets of hippocampal ΔFosB target genes in pilocarpine and APP mice (Stephens et al., 2020), we performed unfiltered GO Network analyses for respective lists of ΔFosB target genes bound in pilocarpine mice (5880 genes), Vehicle-treated controls (759 genes), APP mice (2839 genes), and NTG controls (1933 genes). GO Networks were then filtered to only include GO Terms (nodes that contain target genes involved in a given process) related to immunity and/or neuroprotection. We found significant enrichment (*p* < 0.05) of GO Terms related to immunity and neuroprotection in both vehicle-treated and pilocarpine mice. However, in pilocarpine mice, enriched GO Terms also included disease-related processes such as responses to amyloid-beta, DNA repair, and calcineurin-mediated signaling (Figure 6A). Similar results were obtained when new GO term analyses were performed in APP and NTG mice (Figure 6B).

To visualize classes of cellular function by which seizure-induced ΔFosB might suppress neuroinflammation and maintain neuroprotection regardless of seizure etiology, we generated a new GO Network analysis of the GO Terms related to immunity and neuroprotection (filtered as in Figures 6A, B) that are enriched by the 442 ΔFosB target genes that are shared by Pilocarpine and APP mice (and are not bound in respective controls). The immune- and neuroprotection-focused GO Network was broadly categorized into five key functional domains: Immune Cell and Cytokine Signaling, Debris and Toxin Clearance, DNA Repair, Cell Death, and Oxidative Stress (Figure 6C; gene lists for highlighted GO Terms are in Table 1). A full listing of the immunity and neuroprotection-related GO Terms depicted in the GO Network in Figure 6C is provided in Supplementary Table 2.

**TABLE 1:** Gene ontology (GO) biological processes related to immunity and neuroprotection that are enriched in hippocampal ΔFosB target genes in Pilo and APP mice.

Select gene ontology (GO) terms		Annotated ΔFosB target genes (of 442 shared by Pilo and APP mice)
IMMUNE CELL AND CYTOKINE SIGNALING	Calcineurin-NFAT signaling cascade	*Fhl2, Nfatc1, Rcan1*
	Innate immune response	*A2m, Cx3cr1, Defb8, Fer, Hmgb1, Lgr4, Lsm14a, Ly86, Nlrc5, Oxr1, Ppp6c, Rps19, Trim41*
	Negative regulation of complement activation	*A2m*, ***Cd46***
	Negative regulation of immune system process	*A2m*, ***Cd46****, Cx3cr1, Cxcl12, Dlg5, Fer*, ***Gal****, Gpr137b, Grem1, Hmgb1*, ***Lrfn5****, Nlrc5*, ***Plcb1****, Rps19, Sox9, Tnfrsf21*
	Myeloid cell activation involved in immune response	*Cx3cr1, Fer, Gab2, Hmgb1, Lat, Mrgprb4, Mrgprb5*
DEBRIS AND TOXIN CLEARANCE	Amyloid-beta clearance by cellular catabolic process	*Mme (neprilysin)*
	Cellular glucuronidation	*Ugt1a10, Ugt1a6b, Ugt1a7c, Ugt1a9*
	Protein quality control for misfolded or incompletely synthesized proteins	*Fbxl17, Ube2w*
	Regulation of amyloid-beta formation	*Rtn1, Slc2a13*
	Regulation of establishment of endothelial barrier	***Plcb1*** *, S1pr2*
DNA REPAIR	Cellular response to UV	*Cdc25a, Chek1, Mme, Usp28*
	DNA damage-induced protein phosphorylation	*Chek1*
	Histone H2A acetylation	*Epc1, Mbtd1*
	Negative regulation of double-strand break repair	*Parpbp, Trip12*
	Negative regulation of response to DNA damage stimulus	*Cxcl12, Parpbp, Trip12*
CELL DEATH	B cell apoptotic process	*Slc39a10, Tnfrsf21*
	Hippocampal neuron apoptotic process	*Cx3cr1*
	Negative regulation of hydrogen peroxide-mediated cell death	*Met*
	Oligodendrocyte apoptotic process	*Tnfrsf21*
	Positive regulation of apoptotic process	*Bmpr1b*, ***Gal****, Hmgb1, Inhba, Jmy, Melk, Zmat3*
OXIDATIVE STRESS	Negative regulation of response to oxidative stress	*Met, Oxr1*
	Thioredoxin peroxidase activity	*Selenof*

Bolded gene = implicated in epilepsy (Wang et al., 2017).

## Discussion

In the current study, by using a newly developed specific ΔFosB inhibitor (shRNA) we showed that seizure-induced ΔFosB in the pilocarpine mouse model is part of a homeostatic mechanism that protects the epileptic brain from further deterioration. More specifically, increased ΔFosB activity supports “protective” mossy fiber adaptations, maintains granule cell positioning, and limits neuroinflammatory responses. Furthermore, we recapitulated similar findings (adaptation of mossy fibers and neuroinflammation) in APP mice with recurrent seizures using a previously established inhibitor of ΔFosB activity, a mutant variant of JunD (ΔJunD) that can act in dominant negative fashion. Together, these results demonstrate that ΔFosB exerts critical neuroprotective effects in a seizure etiology-independent manner, indicating that common modes of gene expression can be engaged in distinct neurological disorders accompanied by seizures.

### A balance between neuroprotection and neuroplasticity

We previously demonstrated that seizure-induced ΔFosB accumulation in the DG occurs in both patients and mouse models of Alzheimer’s disease, and that the magnitude of ΔFosB expression corresponded with the magnitude of cognitive impairment (Corbett et al., 2017; You et al., 2017). Those results indicated that ΔFosB may drive cognitive decline, particularly because blockade of ΔFosB activity for several weeks improved spatial memory in APP mice. Indeed, we found that ΔFosB bound to a number of plasticity-related genes whose suppression was directly linked to memory deficits, including cFos and calbindin (Corbett et al., 2017; You et al., 2017; Stephens et al., 2020). However, it is notable that for these gene targets, suppression of their expression is not only linked to deficits in hippocampal function, but also with neuroprotective programs that reduce long-term excitotoxicity in chronic situations (Molinari et al., 1996; Nagerl et al., 2000; Fleischmann et al., 2003; Palop et al., 2003; Calais et al., 2013), suggesting that the actions of ΔFosB drive neuroprotection in chronic conditions. Indeed, prolonged (>1 month) blockade of ΔFosB in APP mice worsened seizures and memory (unpublished observations), consistent with an overall long-term neuroprotective role for ΔFosB. Our findings therefore suggest that seizure-induced ΔFosB may exert neuroprotection at the cost of limiting plasticity, and highlight possible pathways by which it may do so.

### ΔFosB protein preserves hippocampal architecture in the seizing brain

One of the remarkable observations made in the present study is the hippocampal granule cell dispersion detected in the mouse pilocarpine model of mTLE following down-regulation of ΔFosB. While this phenomenon is part of the histopathological features of patients with intractable TLE (Houser, 1990) and is most prominent in the mouse intrahippocampal kainic acid model of mTLE (Bouilleret et al., 1999; Suzuki et al., 2000), it is rarely described in the rodent pilocarpine model (Jagirdar et al., 2016; Moura et al., 2021). Our results indicate that the downregulation of ΔFosB triggered granule cell dispersion, a morphological rearrangement that is typically not observed in the mouse pilocarpine model, pinpointing a key role for ΔFosB in maintaining the positioning of granule cells within the granule cell layer.

One mechanism by which ΔFosB may exert such strong influence on granule cell position is through epigenetic regulation of *Reln*, the gene that encodes the Reelin protein, which we previously found in a ChIP-seq study to be preferentially bound by ΔFosB in pilocarpine mice (Stephens et al., 2020). Previous evidence in the literature supports the role of Reelin in granule cell dispersion related to TLE (Leifeld et al., 2022). Reelin is a secreted glycoprotein present in the extracellular matrix that acts as a stop signal for neuronal migration during development (Tissir and Goffinet, 2003) but its function in adult hippocampus is not well studied. Reelin expression decreases after an epileptogenic brain insult and blocking its function in naïve mice promotes granule cell dispersion (Haas et al., 2002; Gong et al., 2007). Furthermore, exogenous supply of Reelin can prevent granule cell dispersion after an epileptogenic brain insult (Haas and Frotscher, 2010). In line with these findings, the granule cell dispersion seen here in the mouse pilocarpine model corresponded with a dramatic decrease in Reelin expression, especially in the areas surrounding the hippocampal fissure including the stratum lacunosum moleculare and the upper third of the molecular layer of the dentate gyrus. The diffuse Reelin immunostaining in the neuropil of these areas support the idea that Reelin may be secreted from terminals of local inhibitory interneurons expressing Reelin or from afferent axon terminals of the perforant pathway that originates from Reelin-expressing neurons of the entorhinal cortex (Pesold et al., 1998). Secreted Reelin may contribute to the formation of neuronal circuits in the adult brain by the use of mechanisms similar to those of embryonic development (Tissir and Goffinet, 2003). Thus, ΔFosB, by maintaining expression of Reelin in the neuropil surrounding the dentate gyrus, might maintain integrity of neuronal circuits by stopping migration of granular cells in a given direction during epileptic conditions.

Notably, although we previously demonstrated that APP mice exhibit reduced Reelin expression in the dentate gyrus (Chin et al., 2007), the magnitude of reduction was less robust than that exhibited in the pilocarpine-treated mice with suppression of ΔFosB in the current study. The subtle reduction in Reelin in APP mice was not associated with granule cell dispersion, which may reflect the observation that the seizures exhibited by APP mice are lower in frequency and severity (1–3 seizures per week, primarily non-convulsive) relative to those induced after pilocarpine induced-SE. These findings support the hypothesis that the magnitude of ΔFosB expression and the neuroprotective pathways engaged in conditions with seizures are calibrated to the level of neuroprotection required.

Another consistent histopathological finding in patients and animal models with TLE is the increased expression of NPY and SV2C in MF (Schmeiser et al., 2017a; Freiman et al., 2021). In our study, NPY and SV2C immunostaining revealed the MF pathway consisting of axons projecting from the granule cell layer of the DG to the CA3 area, several months after pilocarpine induced-SE in mice. Given that NPY inhibits synaptic transmission at mossy fiber synapses on glutamatergic CA3 pyramidal cells (Klapstein and Colmers, 1993), the increased expression of NPY in MF during epileptic conditions may provide an adaptative and protective mechanism against seizure development. This hypothesis is currently used in the field as a basis for exploiting NPY in gene therapy for epilepsy (Cattaneo et al., 2020). Interestingly, we found that downregulation of ΔFosB in the dorsal hippocampus during the chronic phase of epilepsy after pilocarpine-induced SE decreased NPY staining in the dentate gyrus. The concomitant absence of SV2C staining in the same area suggests that MF underwent degeneration. This loss of MF is also observed in mTLE patients and can be driven by neuronal death in CA3 area (Schmeiser et al., 2017b), a phenomenon that we also observe in Figure 2A (8 weeks after reduction of ΔFosB). We found a similar attenuation of NPY expression in MF in APP mice in which ΔFosB signaling was blocked, supporting the hypothesis that ΔFosB is required for the protective *de novo* expression of NPY in the MF pathway that occurs in distinct conditions with recurrent seizures. Notably, a decrease in MF density can also be observed in the hippocampus of individuals with schizophrenia and in animal models of schizophrenia, and is believed to contribute to behavioral abnormalities found in the disease (Ibi et al., 2020). The decrease in MF density corresponds with a Reelin deficit in the dentate gyrus of the hippocampus in an animal model of schizophrenia, and both behavioral abnormalities and MF deficits can be rescued by delivery of exogenous Reelin into the dentate gyrus of the hippocampus (Ibi et al., 2020), supporting a role of Reelin in the remodeling of MF during disease progression of neurodevelopmental disorders. It is plausible that a similar pathophysiological mechanism involving a Reelin deficit in the dentate gyrus causes the MF loss that we detect in the epileptic brain following ΔFosB downregulation. This possibility is supported by our present results, in which ΔFosB downregulation induced a striking decrease in Reelin expression in the dentate gyrus of the hippocampus from epileptic mice. Altogether, these results indicate that ΔFosB plays an important role in maintaining the adaptative MF pathway in place under epileptic conditions, perhaps through modulation of the Reelin signaling pathway.

### ΔFosB protein attenuates neuroinflammation in the seizing brain

In this study, consistent with previous reports (Mazzuferi et al., 2012; Clasadonte et al., 2013; Wang et al., 2019), a robust neuroinflammation, characterized by astrocytic activation, was detected in the hippocampus of pilocarpine treated mice. Strikingly, downregulation or inhibition of ΔFosB led to an increase in astrocyte and microglia activation in seizing mice regardless of seizure etiology. From our experimental design it is difficult to address whether increased inflammation precedes (directly related to ΔFosB activity) or is secondary to the dramatic changes in hippocampal cytoarchitecture. In a focal mouse model of mTLE, increased astrogliosis or microgliosis precede granule cell dispersion (Pernot et al., 2011). Furthermore, more progressive granule cell dispersion correlates with increased GFAP-positive fiber density (Heinrich et al., 2006), which is in alignment with findings in mTLE patients (Fahrner et al., 2007). Results from our bioinformatic analysis suggest that ΔFosB via its downstream targets like Cxcl12 (Stephens et al., 2020), a chemokine with confirmed role in epilepsy (Song et al., 2016; Zhou et al., 2017; Xu et al., 2019) could suppress further worsening of neuroinflammation in epileptic brain but we cannot rule out a possibility that increased gliosis is a consequence of mossy fiber degeneration or granule cell dispersion or combination of these processes.

Based on data acquired from pilocarpine mouse models and knowledge from the literature, we propose a molecular model in which ΔFosB directly (Stephens et al., 2020), or indirectly through multiple pathways (McClung and Nestler, 2003; You et al., 2018; Lardner et al., 2021), regulates expression of Reelin. This regulation, in turn, sustains the protective actions of mossy fibers and maintains granule cells in their correct position in the epileptic brain. In conclusion, our study indicates that ΔFosB protects the brain from further deterioration during seizures, regardless of seizure etiology. Moreover, we have developed a novel ΔFosB-specific inhibitor that can be utilized by the broader scientific community.

## Data availability statement

Publicly available datasets were analyzed in this study. This data can be found here: https://www.ebi.ac.uk/biostudies/arrayexpress/studies/E-MTAB-8954.

## Ethics statement

The animal study was approved by the Ethical Committee of the High technology Animal Platform, University Grenoble Alpes (experiments at SynapCell); local Ethics Committee/according to Belgian law (experiments at UCB Biopharma SRL); Institutional Animal Care and Use Committee of Baylor College of Medicine (experiments at Baylor College of Medicine). The study was conducted in accordance with the local legislation and institutional requirements.

## Author contributions

JCl: Formal analysis, Investigation, Methodology, Project administration, Visualization, Writing – original draft, Writing – review and editing. TD: Investigation, Methodology, Writing – review and editing. GS: Formal analysis, Investigation, Methodology, Visualization, Writing – original draft, Writing – review and editing. GM-C: Formal analysis, Investigation, Methodology, Project administration, Visualization, Writing – original draft, Writing – review and editing. P-YC: Investigation, Writing – review and editing. MB: Investigation, Methodology, Writing – review and editing. AF: Investigation, Writing – review and editing. JCh: Conceptualization, Funding acquisition, Project administration, Supervision, Writing – original draft, Writing – review and editing. MR: Conceptualization, Formal analysis, Project administration, Supervision, Writing – original draft, Writing – review and editing.

## Supplementary material

Supplementary Table 1 List of primary and secondary antibodies used for immunohistochemistry.

Supplementary Table 2 Full listing of GO Terms represented in network nodes in Figure 6C.

Supplementary Figure 1 Development of specific ΔFosB inhibitor. **(A)** Selection of ΔFosB and FosB transcripts together with sequences that are targeted by developed shRNA (highlighted in brown and red). As shown, only ΔFosB mRNA can be targeted by developed shRNA. Relative gene expression analysis by qPCR of **(B)** ΔFosB and **(C)** FosB transcripts, 4 weeks after AAV-Neg shRNA or AAV-ΔFosB shRNA were injected in the dorsal hippocampus. Statistical test: ANOVA followed by Tukey’s *post-hoc* test (**p* < 0.05; ***p* < 0.01; ns: non-significant; *n* = 6–7).

Supplementary Figure 2 Measuring convulsive seizures in pilocarpine mouse model **(A)** Average number of seizures per day in epileptic mice 2–4 weeks (**left** panel) and from 6 to 8 weeks (**right** panel) after injection of vehicle, AAV-Neg shRNA or AAV-ΔFosB shRNA in the dorsal hippocampus. **Left** panel: *n* = 6 animals per group; non-parametric one-way ANOVA (Kruskal-Wallis test); *p* > 0.05. **Right** panel: *n* = 5–6 animals per group; non-parametric one-way ANOVA (Kruskal-Wallis test); *p* > 0.05). **(B)** Average duration of seizures in epileptic mice 2–4 weeks (**left** panel) and 6–8 weeks (**right** panel) after injection of vehicle, AAV-Neg shRNA or AAV-ΔFosB shRNA in the dorsal hippocampus. **Left** panel: *n* = 6 animals per group; non-parametric one-way ANOVA (Kruskal-Wallis test); *p* > 0.05. **Right** panel: *n* = 5–6 animals per group; non-parametric one-way ANOVA (Kruskal-Wallis test); *p* > 0.05). **(C)** Proportion of seizures of stage 3, 4, and 5 in epileptic mice 2–4 weeks (**left** panel) and 6–8 weeks (**right** panel) after injection of vehicle, AAV-Neg shRNA or AAV-ΔFosB shRNA in the dorsal hippocampus. **Left** panel: 105 seizures from 6 animals for vehicle group, 71 seizures from 6 animals for AAV-Neg shRNA group, 229 seizures from 6 animals for AAV-ΔFosB shRNA group, chi-square contingency test; *p* > 0.05. **Right** panel: 119 seizures from 6 animals for vehicle group, 171 seizures from 6 animals for AAV-Neg shRNA group, 116 seizures from 5 animals for AAV-ΔFosB shRNA group, chi-square contingency test; *p* > 0.05. Pooled data are shown as mean ± SEM. **(D)** Examples of ΔFosB protein analyses by western blotting from pilocarpine treated mice injected with AAV-Neg shRNA or AAV-ΔFosB shRNA showing ΔFosB protein decrease at 4 and 8 weeks after AAV treatment.

Supplementary Figure 3 Immunohistochemistry for Prox1 and c-Fos in the hippocampus of mTLE mouse model. **(A)** Prox1 is primarily expressed in the granule cells of the dentate gyrus and was used to confirm the cellular phenotype of the cells that are dispersed in the hippocampus of the pilocarpine treated mice at 8 weeks. The morphology of the granular layer of the dentate gyrus was markedly affected at 8 weeks with the ΔFosB shRNA. **(B)** c-Fos was used as a marker of neuronal activity in the hippocampus and immunoreactive signal was quantified in the dorsal part of the hippocampus at **(C)** 4 weeks after vehicle or AAV delivery. Data are expressed as mean with standard error of the mean (SEM). Statistical test: ANOVA followed by Tukey’s *post-hoc* test (**p* < 0.05; ***p* < 0.01; *****p* < 0.0001; ns: non-significant). Scale bars = 500 μm.
