# Supplementary material for: ΔFosB is part of a homeostatic mechanism that protects the epileptic brain from further deterioration
Source: Front Mol Neurosci. 2024 Jan 12;16:1324922. doi: 10.3389/fnmol.2023.1324922 (PMC10810990; doi:10.3389/fnmol.2023.1324922)
Supplement: Supplementary Table 1 — List of primary and secondary antibodies used for immunohistochemistry. [file Table_2.DOCX]

| Primary antibody | Supplier | Host/Source | Reference | Dilution |
| --- | --- | --- | --- | --- |
| c-Fos | Synaptic Systems | Rabbit | 226 008 | 1:1,500 |
| DeltaFosB | Cell Signaling | Rabbit | 14695 | 1:1,500 |
| GFAP  (mTLE mice) | Synaptic Systems | Chicken | 173 006 | 1:4,000 |
| GFAP (APP mice) | Chemicon | Rabbit | AB5804 | 1:20,000 |
| GFP, Alexa Fluor 488 conjugated | Chromotek | Alpaca | Gb2AF488 | 1:2,000 |
| Iba1 (mTLE mice) | Abcam | Rabbit | ab178846 | 1:4,000 |
| Iba1 (APP mice) | Wako | Rabbit | 019-19741 | 1:5,000 |
| NeuN, Alexa Fluor 568 conjugated | Abcam | Rabbit | ab207282 | 1:4,000 |
| Neuropeptide Y | Abcam | Rabbit | ab30914 | 1:1,500 for fluor.  1:50K for DAB |
| Prox1 | Abcam | Rabbit | ab199359 | 1:4,000 |
| Reelin | R&D Systems | Goat | AF3820 | 1:1,500 |
| SV2C | ThermoFisher Scientific | Rabbit | PA5-59290 | 1:4,000 |

| Secondary antibody | Supplier | Host/Source | Reference | Dilution |
| --- | --- | --- | --- | --- |
| Anti-Chicken IgY (H+L), Alexa Fluor 555 conjugated | Thermofisher Scientific | Goat | A32932 | 1:1,000 |
| Anti-Rabbit IgG (H+L), Alexa Fluor 647 conjugated | Thermofisher Scientific | Goat | A32733 | 1:1,000 |
| Anti-Rabbit IgG (H+L), Alexa Fluor Plus 555 conjugated | Thermofisher Scientific | Donkey | A32794 | 1:1,000 |
| Anti-Goat IgG (H+L), Alexa Fluor Plus 647 conjugated | Thermofisher Scientific | Donkey | A32849 | 1:1,000 |
| Goat anti-rabbit, biotinylated | Vector | Goat | BA-1000 | 1:200 |

**Supp. Table 1:** **List of primary and secondary antibodies used for immunohistochemistry**
