# Supplementary material for: ΔFosB is part of a homeostatic mechanism that protects the epileptic brain from further deterioration
Source: Front Mol Neurosci. 2024 Jan 12;16:1324922. doi: 10.3389/fnmol.2023.1324922 (PMC10810990; doi:10.3389/fnmol.2023.1324922)
Supplement: Supplementary Table 2 — Full listing of GO Terms represented in network nodes in Figure 6C. [file Table_3.DOCX]

**Supp. Table 2. Full listing of GO Terms represented in network nodes in Figure 5C**

**Immune Cell and Cytokine Signaling**

Calcineurin-NFAT signaling cascade, B-1a B cell differentiation

Dendritic cell differentiation, myeloid dendritic cell differentiation, plasmacytoid dendritic cell activation, positive regulation of toll-like receptor 2, positive regulation of toll-like receptor 9, positive regulation of myeloid progenitor cell differentiation, negative regulation of apoptotic cell clearance (node in *Debris and Toxin Clearance*), positive regulation of mismatch repair (node in *DNA Repair*)

Interleukin-12-mediated signaling pathway, interleukin-15-mediated signaling pathway, CD24 biosynthetic process, monocyte extravasation

Kit signaling pathway, diapedesis, Fc-epsilon receptor signaling pathway

MHC class 1 biosynthetic process, positive regulation of interferon-gamma-mediated signaling pathway, type I interferon pathway

Microglial cell mediated cytotoxicity, positive regulation of microglial cell migration, chronic inflammatory response to non-antigenic stimulus, positive regulation of I-kappaB phosphorylation, host-mediated regulation of intestinal microbiota composition, hippocampal neuron apoptotic process (node in *Cell Death*)

Myeloid cell activation involved in immune response, myeloid leukocyte activation, mast cell activation, mast cell degranulation, myeloid leukocyte mediated immunity

Negative regulation of interleukin-13 production, negative regulation of interleukin-5 production, oligodendrocyte apoptotic process (node in *Cell Death*)

*Singlet GO Terms:* macrophage activation involved in immune response, leukocyte proliferation, dendritic cell proliferation, lymphocyte mediated immunity, positive regulation of B cell receptor signaling pathway, myeloid cell homeostasis, myeloid cell differentiation, chemokine (C-X-C) motif ligand 12 signaling pathway, negative regulation of leukocyte migration, interleukin-10 production, C-C chemokine receptor activity, negative regulation of complement activation, innate immune system process, negative regulation of immune system process, positive regulation of cytokine production

**Debris and Toxin Clearance**

Amyloid-beta metabolic process, regulation of amyloid-beta formation, amyloid-beta clearance by cellular catabolic process, neuropeptide processing, substance P catabolic process, bradykinin catabolic process, cellular response to UV-B (node in *DNA Repair*)

Long-chain fatty acid import into peroxisome, very long-chain fatty acid import into peroxisome, cellular lipid metabolic process, regulation of establishment of endothelial barrier, sphingosine-1-phosphate receptor activity

Negative regulation of apoptotic cell clearance (not singlet; connected to nodes in *Immune Cell and Cytokine Signaling*)

Xenobiotic transport across blood-brain barrier, xenobiotic transmembrane transporter activity, toxin transmembrane transporter activity

*Singlet GO Terms:* RIG-I signaling pathway, 3’-5’-exoribonuclease activity, regulation of protein stability, protein quality control for misfolded or incompletely synthesized proteins, protein repair, cellular glucuronidation

**DNA Repair**

Cellular response to UV-B (connected to nodes in *Debris and Toxin Clearance*), cellular response to UV

DNA damage induced protein phosphorylation, histone kinase activity (H3-T11 specific), regulation of transcription from RNA polymerase II promoter in response to UV-induced DNA damage

Negative regulation of double-strand break repair, negative regulation of response to DNA damage stimulus

Positive regulation of mismatch repair (not singlet; connected to nodes in *Immune Cell and Cytokine Signaling*)

Histone H2A acetylation, positive regulation of double-strand break repair via homologous recombination

*Singlet GO Terms:* none

**Cell Death**

Hippocampal neuron apoptotic process (not singlet; connected to nodes in *Immune Cell and Cytokine Signaling*)

Oligodendrocyte apoptotic process (connected to nodes in *Immune Cell and Cytokine Signaling*), B cell apoptotic process

Negative regulation of hydrogen peroxide mediated cell death (not singlet; connected to node in *Oxidative Stress*)

*Singlet GO Terms:* mitochondrial genome maintenance, positive regulation of apoptotic process, ceramide-1-phosphate transfer activity

**Oxidative Stress**

Negative regulation of response to oxidative stress (not singlet; connected to node in *Cell Death*)

*Singlet GO Terms:* thioredoxin peroxidase activity
