# Supplementary figures and images for: ΔFosB is part of a homeostatic mechanism that protects the epileptic brain from further deterioration
Source: Front Mol Neurosci. 2024 Jan 12;16:1324922. doi: 10.3389/fnmol.2023.1324922 (PMC10810990; doi:10.3389/fnmol.2023.1324922)

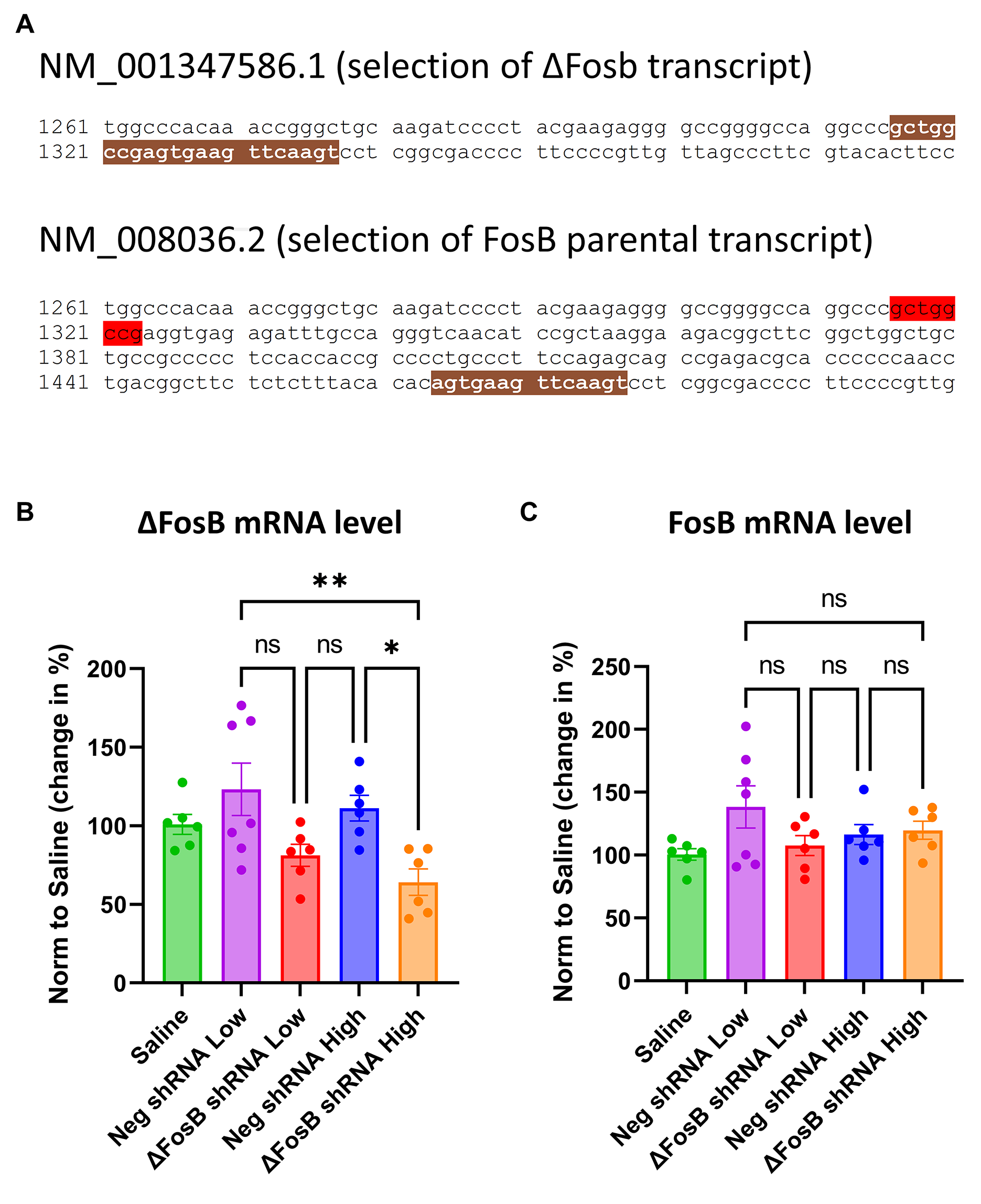

Supplement: Supplementary Figure 1 — Development of specific ΔFosB inhibitor. (A) Selection of ΔFosB and FosB transcripts together with sequences that are targeted by developed shRNA (highlighted in brown and red). As shown, only ΔFosB mRNA can be targeted by developed shRNA. Relative gene expression analysis by qPCR of (B) ΔFosB and (C) FosB transcripts, 4 weeks after AAV-Neg shRNA or AAV-ΔFosB shRNA were injected in the dorsal hippocampus. Statistical test: ANOVA followed by Tukey’s post-hoc test (*p < 0.05; **p < 0.01; ns: non-significant; n = 6–7). [file Image_1.TIF]

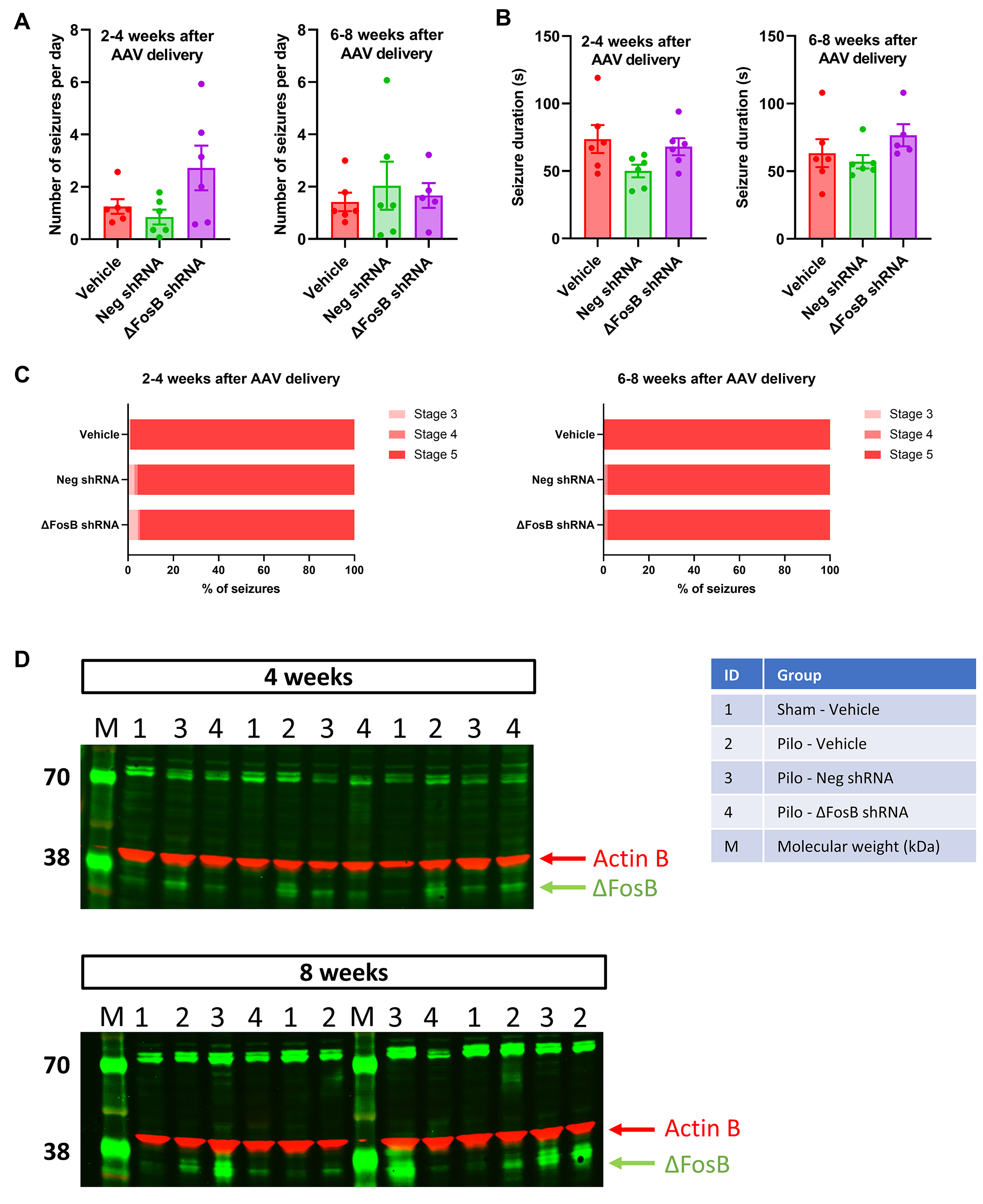

Supplement: Supplementary Figure 2 — Measuring convulsive seizures in pilocarpine mouse model (A) Average number of seizures per day in epileptic mice 2–4 weeks (left panel) and from 6 to 8 weeks (right panel) after injection of vehicle, AAV-Neg shRNA or AAV-ΔFosB shRNA in the dorsal hippocampus. Left panel: n = 6 animals per group; non-parametric one-way ANOVA (Kruskal-Wallis test); p > 0.05. Right panel: n = 5–6 animals per group; non-parametric one-way ANOVA (Kruskal-Wallis test); p > 0.05). (B) Average duration of seizures in epileptic mice 2–4 weeks (left panel) and 6–8 weeks (right panel) after injection of vehicle, AAV-Neg shRNA or AAV-ΔFosB shRNA in the dorsal hippocampus. Left panel: n = 6 animals per group; non-parametric one-way ANOVA (Kruskal-Wallis test); p > 0.05. Right panel: n = 5–6 animals per group; non-parametric one-way ANOVA (Kruskal-Wallis test); p > 0.05). (C) Proportion of seizures of stage 3, 4, and 5 in epileptic mice 2–4 weeks (left panel) and 6–8 weeks (right panel) after injection of vehicle, AAV-Neg shRNA or AAV-ΔFosB shRNA in the dorsal hippocampus. Left panel: 105 seizures from 6 animals for vehicle group, 71 seizures from 6 animals for AAV-Neg shRNA group, 229 seizures from 6 animals for AAV-ΔFosB shRNA group, chi-square contingency test; p > 0.05. Right panel: 119 seizures from 6 animals for vehicle group, 171 seizures from 6 animals for AAV-Neg shRNA group, 116 seizures from 5 animals for AAV-ΔFosB shRNA group, chi-square contingency test; p > 0.05. Pooled data are shown as mean ± SEM. (D) Examples of ΔFosB protein analyses by western blotting from pilocarpine treated mice injected with AAV-Neg shRNA or AAV-ΔFosB shRNA showing ΔFosB protein decrease at 4 and 8 weeks after AAV treatment. [file Image_2.TIF]

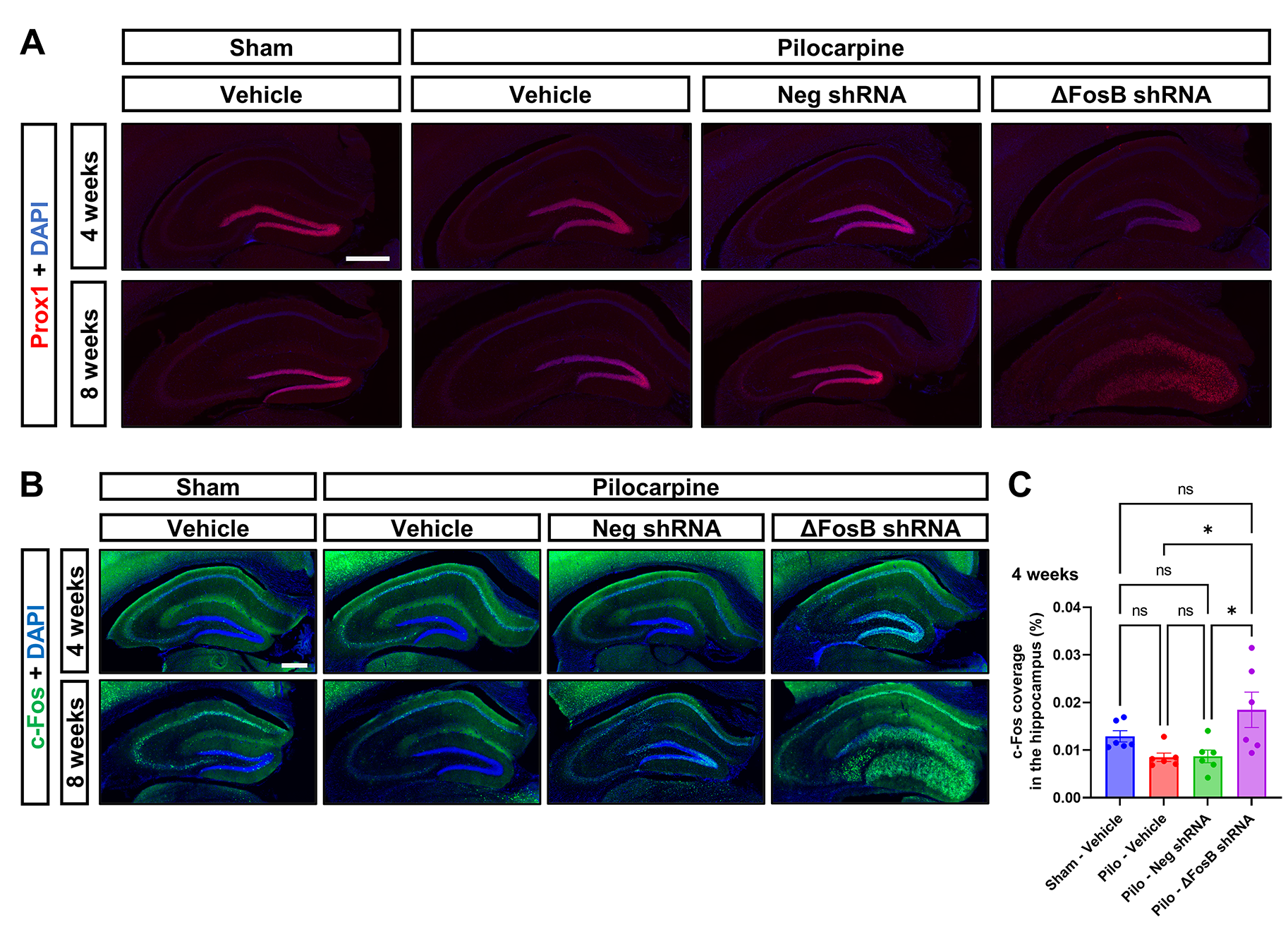

Supplement: Supplementary Figure 3 — Immunohistochemistry for Prox1 and c-Fos in the hippocampus of mTLE mouse model. (A) Prox1 is primarily expressed in the granule cells of the dentate gyrus and was used to confirm the cellular phenotype of the cells that are dispersed in the hippocampus of the pilocarpine treated mice at 8 weeks. The morphology of the granular layer of the dentate gyrus was markedly affected at 8 weeks with the ΔFosB shRNA. (B) c-Fos was used as a marker of neuronal activity in the hippocampus and immunoreactive signal was quantified in the dorsal part of the hippocampus at (C) 4 weeks after vehicle or AAV delivery. Data are expressed as mean with standard error of the mean (SEM). Statistical test: ANOVA followed by Tukey’s post-hoc test (*p < 0.05; **p < 0.01; ****p < 0.0001; ns: non-significant). Scale bars = 500 μm. [file Image_3.TIF]
